# Evaluation of Energy Absorption Capabilities of Polyethylene Foam under Impact Deformation

**DOI:** 10.3390/ma14133613

**Published:** 2021-06-29

**Authors:** Baohui Yang, Yangjie Zuo, Zhengping Chang

**Affiliations:** 1 School of Aeronautics and Astronautics, Sichuan University, 24 Nanyi Section Yihuan Road, Chengdu 610065, China; baohuiyang521@gmail.com; 2 Department of Mechanical Engineering, Northwestern Polytechnical University, 127 Youyi Ave. West, Xi’an 710072, China; chzhping@nwpu.edu.cn

**Keywords:** foam, high strain rate, energy absorption, failure

## Abstract

Foams are widely used in protective applications requiring high energy absorption under impact, and evaluating impact properties of foams is vital. Therefore, a novel test method based on a shock tube was developed to investigate the impact properties of closed-cell polyethylene (PE) foams at strain rates over 6000 s^−1^, and the test theory is presented. Based on the test method, the failure progress and final failure modes of PE foams are discussed. Moreover, energy absorption capabilities of PE foams were assessed under both quasi-static and high strain rate loading conditions. The results showed that the foam exhibited a nonuniform deformation along the specimen length under high strain rates. The energy absorption rate of PE foam increased with the increasing of strain rates. The specimen energy absorption varied linearly in the early stage and then increased rapidly, corresponding to a uniform compression process. However, in the shock wave deformation process, the energy absorption capacity of the foam maintained a good stability and exhibited the best energy absorption state when the speed was higher than 26 m/s. This stable energy absorption state disappeared until the speed was lower than 1.3 m/s. The loading speed exhibited an obvious influence on energy density.

## 1. Introduction

Polymeric foams are widely used in protective applications due to their high energy absorption capability [1,2,3], and the properties of a variety of open-cell and closed-cell foams have been widely studied under quasi-static compression [4,5,6], impact, and high strain rate loading conditions [7,8]. Foam materials and other materials can be combined into composite materials with excellent properties as needed [9,10,11]. For example, when polymer foam material is used as the core of sandwich composite [12,13,14], its impact resistance and energy absorption effect are significantly improved compared with similar materials [15,16]. With the development of the aviation industry, more and more aircraft structures have used polymer foam core sandwich composites. The craft, however, will encounter unexpected loads, such as bird strikes or other objects. In order to improve the impact resistance of aircraft, it is of great significance to study the energy absorption characteristics of polymer foam material.

Many scholars have conducted impact tests on materials, and the foam material exhibited obvious sensitivity to high strain rate [17,18,19]. Luong et al. carried out the Hopkinson pressure bar test on a very thin polyvinyl chloride (PVC) foam. However, because of factors such as the cellular structure and viscoelastic nature of the polymer, the evaluation of elevated strain rate properties of foams is challenging [20,21,22]. However, the standard test methods have some limitations in characterizing foams due to their large elastic strain, viscoelastic material characteristics, and high damage tolerance and the methods need to evolve for such materials [23,24,25].

The impact test of foam with intermediate strain rate loading was explored. The drop weight impact test has been widely used for characterizing the intermediate strain rate impact properties of foams [26,27], where a plate-like specimen is loaded under mid-point impact conditions [28]. This kind of testing allows for observing the extent of damage in the foam with respect to the distance from the impact location [14]. However, a limitation of this method is that the stress–strain diagram cannot be developed because only a small section of the specimen is subjected to direct loading. In the absence of complete fracture, there may be vibrations in the specimen and multiple impacts due to rebound (although air brakes and other methods can be used to minimize such effects). In addition, the results obtained from this kind of impact cannot be compared to those obtained from compression tests due to the difference in the specimen loading configuration. The present work involved a modified drop weight impact instrumentation, where a standard hemispherical impact tup was replaced with a 25 mm diameter flat face tup to load a cylindrical specimen of the same diameter. The experiment allowed for developing the stress–strain diagram for the specimen and computing the energy absorption for comparison with those obtained from quasi-static and high strain rate loading conditions [29,30]. The experimental results of dynamic mechanical tests on foam materials showed that the density and temperature of the material will have a great influence on the mechanical properties and energy absorption effect of foam materials [31,32,33,34].

The impact test of foam with high strain rate loading was also explored. Experiments on the foams show that the strain in the foam is not uniform along the specimen length at high strain rates. The experimental results showed that the stress of the material increases with the increase in strain rate. The observation that the specimen deformation is nonuniform is also supported by finite element analysis simulations, which allowed for dividing the foam behavior into three modes based on strain rates: homogeneous mode, transitional mode, and dynamic mode. A split Hopkinson pressure bar is the most popular experimental method in the study of metal high strain loading. However, since the loading time and loading strain displacement of the split Hopkinson pressure bar have certain limitations, the condition of high strain rate loading for closed cell polymer foam of large thickness cannot be achieved. The results from impact tests are augmented with high strain rate loading results obtained from a shock tube-based test method [35,36,37]. Using a shock tube as a controlled experimental platform is very helpful for determining the high strain rate properties of materials. This test can provide the necessary data for material design. Shock tubes, having a single or a double diaphragm fracture mechanism to generate shock waves, have been widely used in aerodynamics research [38,39]. Shock tubes have also been used to study the effect of the shock wave on the deformation behavior of foams [40]. However, the previous shock tube-based studies were focused on the post-mortem of foams to observe the deformation and failure mechanisms. The shock tube developed in this work provides load–displacement data from the load cell, pressure transducers, and a high-speed camera and allows for comparing the energy absorption capability in other loading conditions. A transparent specimen chamber is constructed to allow for capturing the specimen deformation using a high-speed camera.

This work used a modified drop weight impact tower to conduct the testing of polyethylene (PE) foam at intermediate strain rate compression. The stress–strain curve of the foam at an intermediate strain rate was obtained. The results compared the energy absorption results with the values obtained under quasi-static compression tests. In order to study the energy absorption characteristics of polymer foam under high strain loading, a new method to obtain the energy absorption was found through theoretical derivation. An in-house developed shock tube test platform was used for the testing of PE foam at high strain rate compression. The energy absorption characteristics of PE foam under the impact were analyzed and are discussed.

## 2. Theory Analysis

### 2.1. Deformation Modes and Transitional Compression Velocity

**Figure 1:**
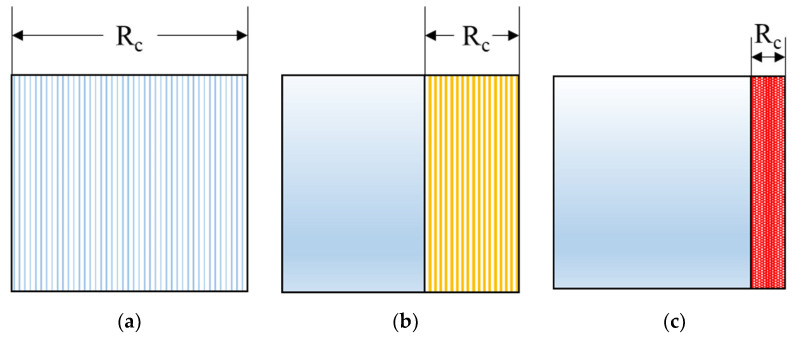
Three deformation modes of foam material under different loading rates [41]. R_c_ represents the compressed part. (**a**) Homogeneous mode: the stress values on the proximal and distal surfaces of the specimen are equal and the strain is distributed uniformly along the specimen length. (**b**) Transitional models: the impacted foam collapsed and the proximal end has not densified. (**c**) Shock mode: the impacted foam rapidly collapsed and densified close to the proximal end.

The specimen deformation becomes progressively more localized as the compressive strain rate is increased and the deformation mode changes from homogeneous to transitional to shock mode, as shown in Figure 1 [41]. In the homogeneous mode, the stress values on the proximal and distal surfaces of the specimen are equal and the strain is distributed uniformly along the specimen length. However, when the compression velocity is large enough, the impacted foam will rapidly collapse and densify close to the proximal end. After sufficient densification, which depends on the strain rate, the stress is transferred to the next layer of cells in the specimen and this phenomenon continues either until the entire specimen is densified or until the strain is applied to the specimen. This effect becomes more pronounced as the compressive strain rate is increased. The stress transfer within the specimen from the proximal to distal end depends on the strain rate and total applied strain.

**Figure 2:**
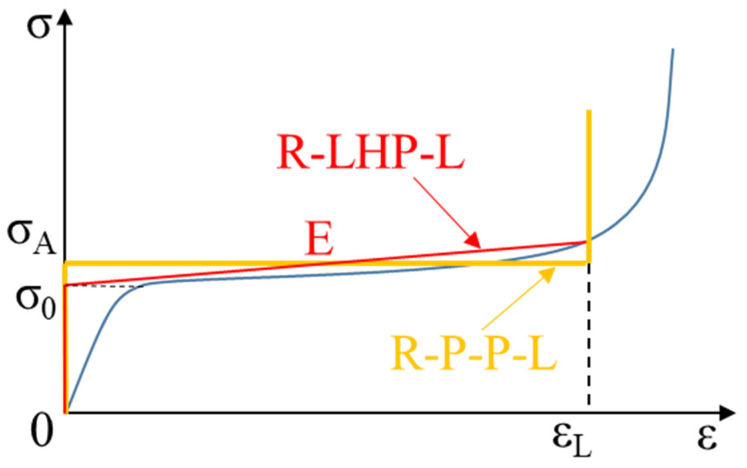
Diagram of the stress σ of foam materials as a function of the strain ε, the green line is R-P-P-L model and the red line is R-LHP-L model [42]. Here, σ_0_ is the yield stress of the material, ε_L_ is the locking strain of the material, E_1_ is the hardening modulus of the material, and σ_pl_ is the plateau stress.

The rigid–linearly hardening plastic–locking (R-LHP-L) model is adopted to judge the three modes [42]. The first critical compression velocity that causes transition from homogeneous mode to transitional mode, *V_c_*_1_, is described by [42]:

(1) $$V_{c1}=\frac{\sigma_{0}}{9\rho_{0}\sqrt{\frac{E_{1}}{\rho_{0}}}}$$

where *σ*_0_ is the yield stress of the material, *ρ*_0_ is the density of the specimen in the original state, and *E*_1_ is the strain hardening modulus of the material (Figure 2).

When the impact stress of *σ_A_* inside the specimen is greater than the compaction stress of the specimen *σ_(εL)_*, the shock mode appears [41,42,43,44]:

(2) $$\sigma_{0}+\frac{\rho_{0}V_{s}^{2}}{\varepsilon_{L}}\geq \sigma_{0}+E_{1}\varepsilon_{L}$$

where *V_s_* is the compression velocity of the foam, ε*_L_* is the densification strain of the foam.

When the inequality above is equal, the second critical velocity is obtained, that is, the critical velocity for the shock mode to transitional mode:

(3) $$V_{c2}=\varepsilon_{L}\sqrt{E_{1}/\rho_{0}}$$

Therefore, when the deformation velocity of the specimen is less than *V_c_*_1_, the specimen is in homogeneous mode. When the deformation velocity of the specimen is greater than *V_c_*_1_ and less than *V_c_*_2_, the specimen is in the transitional mode. When the deformation velocity of the specimen is greater than *V_c_*_2_, the specimen is in shock mode [41].

### 2.2. Energy Absorption

In homogeneous mode, the energy absorption of foam under plastic deformation can be calculated according to the stress–strain curve under quasi-static loading [45,46,47]:

(4) $$Eq=D\int_{0}^{\varepsilon_{L}}\sigma \left(\varepsilon \right)d\varepsilon$$

where *E_q_* is the energy absorption of foam under quasi-static loading, *ε_L_* is the strain entering the densification phase, *σ* is the stress of the foam, *D* is the volume of the specimen.

In the transitional and shock modes, the strain in the foam is not uniform along the specimen length at high strain rates, which makes the calculation of the foam material unreliable. Therefore, we cannot get the strain–stress curve. Therefore, we have to find another way to calculate the energy absorption. According to [41], the impact energy can be divided into deformation energy and other energy loss, and can be given by:

(5) $$E_{i}=E_{s}+E_{w}\;$$

where *E_s_* and *E_w_* are the energy absorption of foam under the high strain rate loading and the other energy loss, respectively.

According to the rigid–plastic–locking (R-P-P-L) model, the dynamic crushing stress *σ_A_* at the proximal end is derived as [43,48,49,50]:

(6) $$\sigma_{A}=\sigma_{0}+\frac{\rho_{0}V_{s}^{2}\left(t\right)}{\varepsilon_{l}}\;\;$$

The foam energy absorption is calculated by:

(7) $$E_{s}=D\int_{0}^{t}\left(\sigma_{A}\left(t\right)\varepsilon_{l}+\frac{1}{2}\rho_{0}V_{s}^{2}\right)dt\;$$

The impact energy of the incident wave in the shock tube can be calculated by [51]:

(8) $$E_{i}=\overset{t}{\underset{0}{\iint }}p_{s}v_{s}dsdt$$

where *E_i_* is the impact energy of the incident wave.

## 3. Experimental Testing

### 3.1. Specimen Details

**Figure 3:**
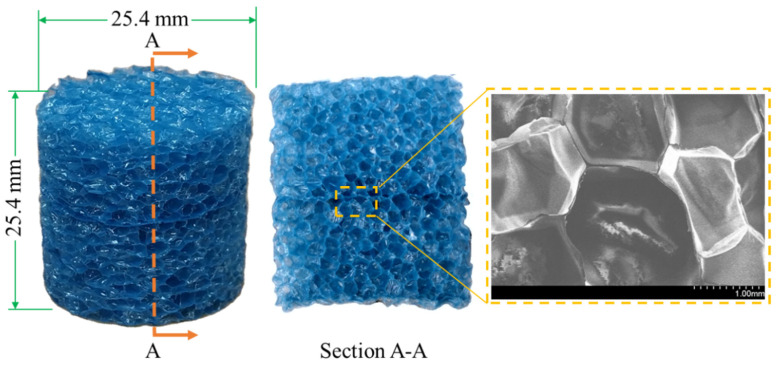
The structure of the PE foam specimen. The density is 27.2 kg/m^3^, the average diameter and the wall thickness of the cell are 1.49 mm and about 0.017 mm, respectively. Both the diameter and height of the specimens are 25.4 mm.

Closed-cell polyethylene (PE) foam (Pregis, Aurora, IL, USA) with a density of 27.2 kg/m^3^ was selected, as shown in Figure 3. The average diameter of the cells and the wall thickness were 1.49 mm and about 0.017 mm, respectively. Cylindrical specimens of 25.4 mm in diameter and height were used for testing. The Poisson’s ratio of the material was found to range from 0.15 to 0.78.

### 3.2. Experimental Methods

**Figure 4:**
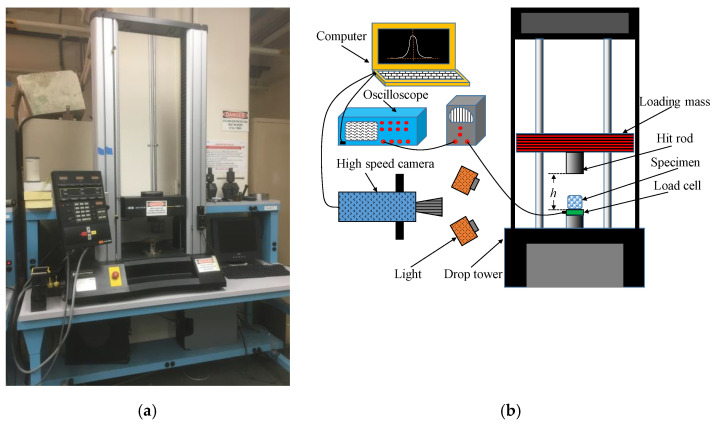
The experimental testing platform for foam materials: (**a**) the hydraulic testing machine corresponding to the quasi-static strain rate test; (**b**) the drop tower testing machine corresponding to the intermediate strain rate test.

The compression tests were conducted under three different loading conditions. The Instron 4467 (Instron, Norwood, MA, USA) universal test machine with a 30 kN load cell was used for quasi-static compression tests, as shown in Figure 4a. Nine specimens were tested under the quasi-static test (QSST), which were divided into three groups. The strain rates of these QSSTs were set at 10^−3^ s^−1^, 10^−2^ s^−1^, and 10^−1^ s^−1^.

The intermediate strain rate compression tests were conducted on a modified Dynatup 9200 series drop weight impact tower, as shown in Figure 4b. The compressive force released during the impact between the impactor and specimen was measured by a dynamic load cell (PCB 208BC03, PCB, Depew, NY, USA), which was mounted under the specimen. The signal of the load cell was amplified and transmitted to an oscilloscope (Tektronix TDS 2014B, Tektronix, Inc., Beaverton, OR, USA) before being captured by a computer. A high-speed camera (NAC MEMRECAM HX-5, nac Americas, Ins., Salem, MA, USA) was used to capture the specimen deformation at a frame rate of 4000 Hz. An in-house Matlab code was developed to process the high-speed camera images to measure specimen deformation. The impact mass was 4 kg. The height of the impactor *h* was selected as 76 mm and 102 mm, and three specimens were tested for each one. The impact speed was 1.22 m/s and 1.4 m/s, respectively. The impact energies were 4 J and 3 J, respectively. The strain rates of these drop weight tests were set at 40 s^−1^ and 36 s^−1^, respectively.

**Figure 5:**
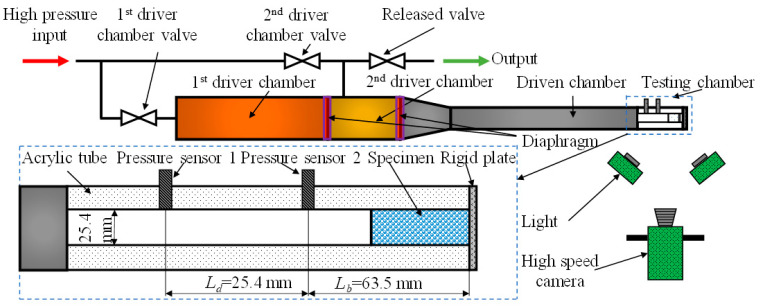
The shock tube experiment platform.

The third group of tests were conducted at high strain rates using a double diaphragm aerodynamic shock tube. The stainless-steel shock tube had two driver chambers and one driven chamber, as shown in Figure 5. The diameters of the driver and driven chambers were 50.8 mm and 25.4 mm, respectively. An incident shock wave was generated after two diaphragms sequentially ruptured because of the difference in pressure set in the two driver chambers. This incident shock wave traveled through the driven chamber and interacted with the specimen placed in a transparent acrylic test chamber. *L_d_* is the distance between two pressure sensors, and *L_b_* is the distance between the second sensor and rigid plate. The pressure in the shock tube was measured by two pressure sensors (PCB 101A06, PCB, Depew, NY, USA) before being recorded by an oscilloscope (Tektronix TDS 2014B, Tektronix, Inc., Beaverton, OR, USA). Five specimens were tested, and their deformation was also captured by the high-speed camera with a frame rate at 100,000 Hz. The incident shock was set at 2.1 Mach.

## 4. Results and Discussion

### 4.1. Stress–Strain Response

**Figure 6:**
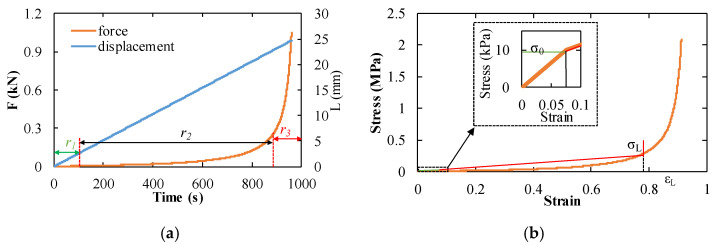
The mechanical response characteristics of foam materials in quasi-static experiments under a strain rate of 10^−3^ s^−1^: (**a**) The diagram of the force and the displacement of foam materials as a function of the time, *r*_1_ is the linear phase, *r*_2_ is the plateau phase, and *r*_3_ is the density phase, the orange line represents the force of the specimen, and the blue line represents the compression displacement of the specimen. Moreover, the whole deformation process of foam exhibited the linear phase, the plateau phase, and the density phase, respectively; (**b**) the diagram of the stress of the foam as a function of the strain, the yield stress σ_0_ is 0.00998 MPa, the locking stress σ_L_ is 0.27 MPa, and the locking strain ε_L_ is 0.78.

Representative force–time and stress–strain curves under QSST loading are shown in Figure 6. The curve shows a long stress plateau that corresponds to the high energy absorption under compression, then the stress rises sharply when entering the densification stage. The end of the stress plateau and onset of densification stage means the cell wall ruptured and the foam microstructure was permanently damaged. This is also a key factor in the foam’s ability to absorb energy. The test was conducted at a strain rate of 10^−3^ s^−1^ calculated from Figure 6a, allowing uniform compression of the foam specimen, as the length L of the specimen and the strain rate of the compression specimen were known. Therefore, the force–time curve can be converted to obtain the stress–strain curve of the specimen, as shown in Figure 6b. According to reference [42], *σ*_0_, *ε_L_*, and *E*_1_ can be obtained from the stress–strain curves.

**Figure 7:**
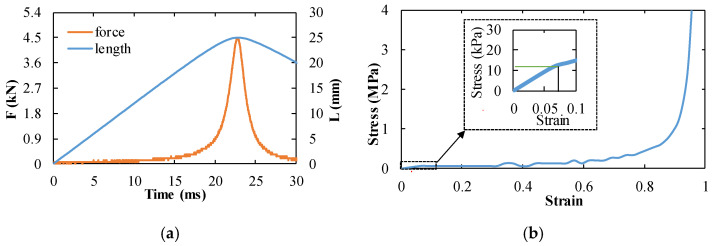
The mechanical response characteristics of the foam in intermediate experiments under a strain rate of 40 s^−1^: (**a**) drop tower test curve corresponding to the displacement, force, and time of the experiment, the orange line represents the force of the specimen, and the blue line represents the compression displacement of the specimen; (**b**) the strain–stress curve of the foam in the drop tower experiments, here, the yield stress is 0.0109 MPa.

In the drop weight experiment, the compressive force and the specimen length were simultaneously measured and are plotted over time in Figure 7a. In this figure, the specimen length steadily decreased at a constant rate while the compressive load increased moderately, and its rate turned to zero at the moment the compressive load reached the maximum value. This means that the specimen deformed at a constant strain rate in the elastic and plateau regions. Consequently, the stress–strain curve is shown in Figure 7b, and its strain rate was calculated over the proportional region of the specimen displacement–time relationship. The intermediate strain rate for this experiment was in the range of 40 s^−1^.

The basic characteristics of the specimen deformation are similar to those at quasi-static loading. Here, a plateau region is followed by a densification stage. However, the plateau region shows significant vibration, as shown in Figure 7a. The deformation at an intermediate strain rate is not expected to be uniform along the specimen length. Hence, local microstructure variations in the foam affect the foam compression response observed in Figure 7b. According to the properties of foam, the plateau vibration represents the collapse activity of the stomatal unit. The magnitude of the vibration is determined by the number of stomatal units that collapse due to this initial cascade effect. In some foams, there is a wide range of stomatal sizes and stomatal wall thicknesses. Once a stomatal unit collapses, the stress is redistributed in the surrounding area and a new equilibrium is reached. The result is a smooth plateau with slow deformation. In the process of rapid deformation, there is not enough time to redistribute the stress, which causes a significant vibration phenomenon.

**Figure 8:**
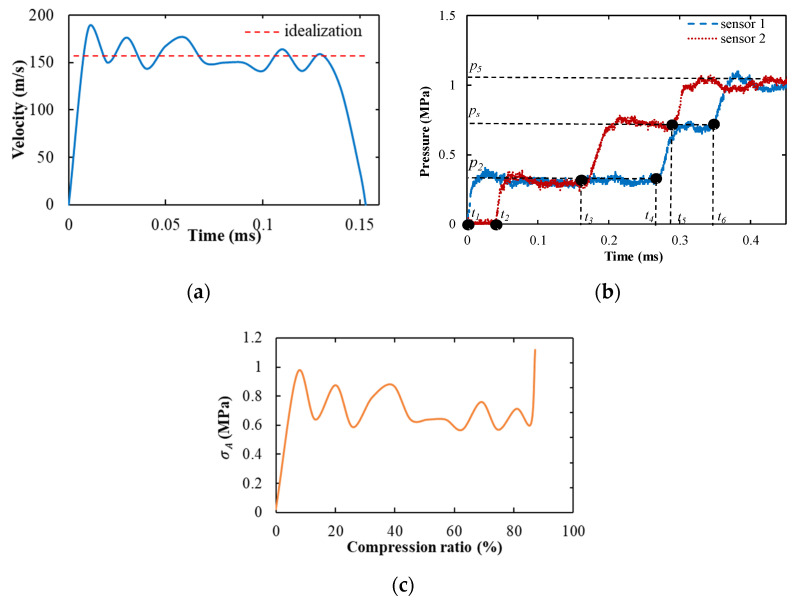
The testing results in the shock tube test: (**a**) the diagram of the velocity of the compression deformation as a function of the time, here, the blue line represents the experiment velocity and the red line represents the idealized velocity, and the average velocity of the compression is about 155 m/s; (**b**) the typical corresponding pressure–time curve of the shock tube, here, the blue line represents the pressure recorded by sensor 1 and the red line represents the pressure recorded by sensor 2, *p_s_* represents the first reflected wave, and *p*_5_ represents the second reflection wave; (**c**) the dynamic crushing stress *σ_A_* as a function of the specimen compression ratio in the shock tube experiment, which was calculated by Equation (6).

The representative shock test results of foam specimens are presented in Figure 8. The deformation velocity (*V_s_*) of the specimen hovered around 155 m/s and the specimen deformation continued for 0.16 ms, as shown in Figure 8a. If the internal strain of the specimen is assumed to be uniform during the compression process, the strain rate of the specimen should be 6100 s^−1^. However, in fact, the internal stress of the specimen was not uniform (this phenomenon will be shown in the next section), so it can be judged that the strain rate of the specimen was greater than 6100 s^−1^, which is a high strain rate deformation. The pressure curve was obtained by the two pressure sensors mounted in the shock tube. The incident shock wave generated by the rupture of the diaphragm propagates in the driver chamber [38,39] and propagates under atmospheric pressure *p*_1_. The pressure of the incident shock front is denoted as *p*_2_ and recorded by two pressure sensors. When the incident wave hits the specimen, the first reflected wave *p_s_* is generated. When the specimen is completely compressed and hits the rigid body, a second reflection wave (*p*_5_) is generated. The pressures behind the shock wave, *p*_2_, *p_s_*, and *p*_5_, all maintain a stable pressure value (see Figure 8b). During the shock wave compression of the specimen, the specimen was subjected to a constant pressure of *p_s_*. The dynamic crushing stress *σ_A_* can be calculated by Equation (6), where *σ*_0_ can be obtained from the quasi-static tests and *V_s_* can be obtained from Figure 8a. The stress–compression ratio curve of the specimen front during the whole compression is presented in Figure 8c. It can be argued that this figure does not present the stress–strain curve for the specimen, because that the specimen deformation behavior at such a high strain rate is not uniform and the strain is preferentially localized close to the specimen front surface.

### 4.2. Failure Mechanism

**Figure 9:**
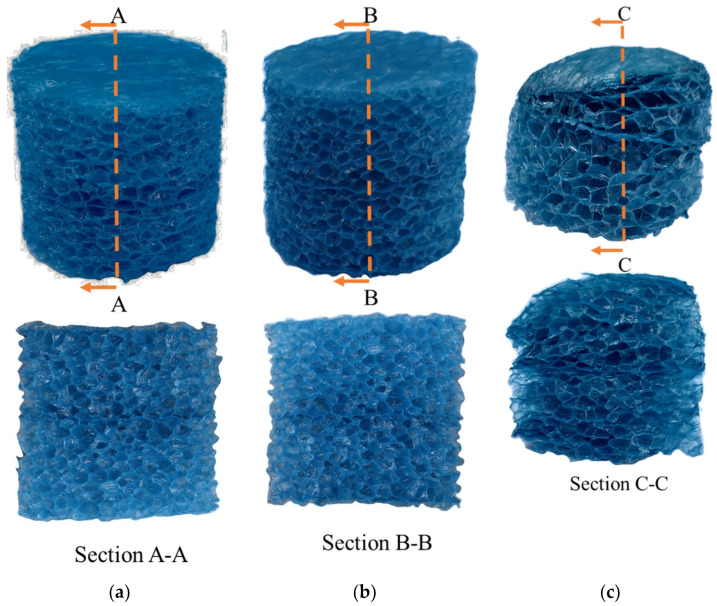
The final deformation of the foam under different loading rates, the bottom figures are the cross section of the specimens cut along the section marked in the top figures: (**a**) specimen failed under the quasi-static loading; (**b**) specimen failed under intermediate strain rate loading; (**c**) specimens failed under high strain rate loading.

A representative set of specimens failed under quasi-static, intermediate, and high strain rate compression are shown in Figure 9a–c, respectively. Although the quasi-static (10^−3^ s^−1^) and intermediate (40 s^−1^) tests were conducted at four orders of magnitude of strain rate, the failure features of specimens are similar, which show that some plastic deformation is not recovered and densified cells scattered in the specimen. However, the shock tested (greater than 6100 s^−1^) partially melts and resolidifies the specimen surface during the high strain rate deformation. The shock wave has a high temperature, which caused this phenomenon. The proximal part of the specimen (the top of the specimen in Figure 9c) shows densification due to the melting and a greater amount of damage than the bottom part. When the specimen is opened, it can be seen that the changes in internal and external appearance are consistent. The cells inside were twisted, and many of the cells had a flat shape. Overall, the damage is nonuniform in the specimen along the length.

These observations show that the specimen failure behavior transitions from uniform to nonuniform compression and the transition zone is greater than the strain rate of 40 s^−1^ tested in this work. The parameters *σ*_0_, *ε_L_*, and *E*_1_ are obtained from the quasi-static curves of the specimen and are substituted into Equations (1) and (3), respectively, to obtain *V_c_*_1_ = 1.3 m/s and *V_c_*_2_ = 26 m/s. It is found that the tup velocity did not reach the condition of transition mode, while the velocity of shock wave reached the condition of shock mode.

**Figure 10:**
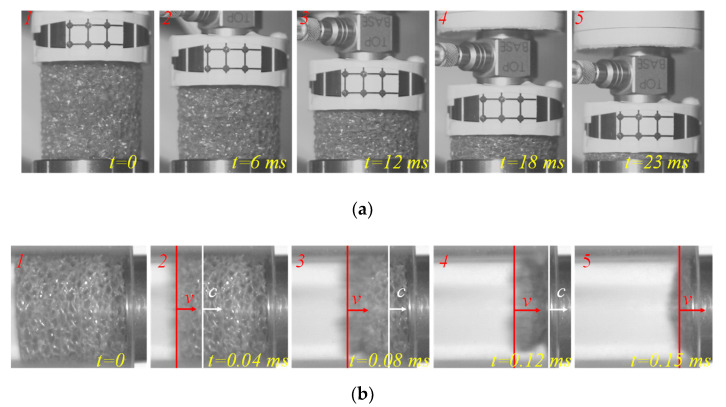
The typical deformation progress of the foam in drop weight impact test and shock tube test: (**a**) drop weight impact test; (**b**) shock tube experiment. Here, *v* represents the velocity of the compression deformation, and *c* represents the impact velocity in the foam.

The high-speed camera images, shown in Figure 10, also provide further evidence. The observations of drop weight impact in Figure 10a show that the center of the specimen has deformed preferentially, not the top part close to the impact. The deformation localization in the center is likely due to the area that triggered the initial failure of a few cells that led to preferential compression. Figure 10b includes the images of the specimen captured by the high-speed camera during shock testing. It can be seen from the picturesthat the overall deformation of the specimen is not uniform from the beginning of the test. The images at 0.04 and 0.08 ms show that the front part of the specimen was much more severely deformed than the back part. The images at 0.12 and 0.15 ms show a very interesting fact. The specimen front takes a concave shape, likely similar to the shape of the shock front at 0.12 ms and then it reverses to a convex shape at 0.15 when the shock reflects from the back part. This convex shape of the specimen is also visible in Figure 10b for the shock loaded specimen, while the quasi-static and intermediate strain rate tested specimens show a flat front. Therefore, the experimental results agree with the theoretical expectation.

### 4.3. Energy Absorption

**Figure 11:**
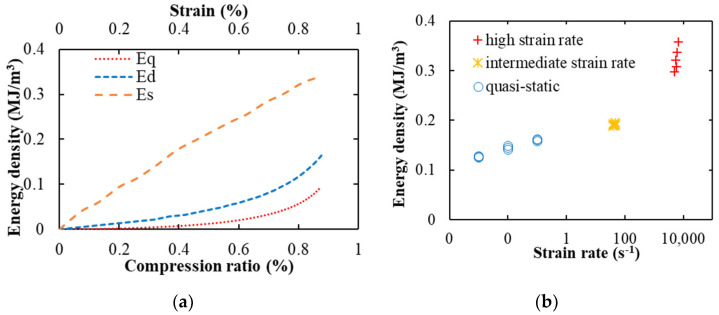
The energy absorption of the foam under the different strain rates: (**a**) the diagram of the energy absorption as a function of the compression ratio, the red line (E_q_) represents the energy absorption of the foam in the hydraulic test, the blue line (E_d_) represents the energy absorption of the foam in the drop weight test, and the orange line (E_s_) represents the energy absorption of foam in the shock tube test; (**b**) the energy absorptions of the foam under the different strain rates, here, the energy absorption value was greatly affected by velocity under the shock mode.

In order to further study the energy absorption state of the specimen under different strain rates, the deformation of the specimen under the quasi-static test and the drop weight test belong to the uniform mode deformation. Therefore, Equation (4) was used to calculate the energy absorption by the foam specimen tested under quasi-static and drop weight tests. The experimental deformation of the shock tube belongs to the shock mode deformation. Combining the specimen velocity curve with Equation (7), the energy absorption of the specimen can be calculated under the shock mode. The energy absorption characteristics of the foam under the three loading conditions are shown in Figure 11a. The energy density was calculated as a function of deformation strain for the quasi-static test and drop weight test, and the energy density was calculated as a function of the compression ratio for the shock mode test. The diagram shows a steady increase in the energy density in the elastic and plateau region. The energy density increases rapidly in the densification region after the compression ratio of the specimen reaches 0.7. The final total compression energy density of the specimen in quasi-static and intermediate strain rate reaches 0.1 MJ/m^3^ and 0.17 MJ/m^3^, respectively. It can be seen from the references that the energy density curves obtained are the same as those obtained in [52,53]. Moreover, the energy density trend under the shock loading condition was different. The specimen did not show any densification effect and the energy density was linear in the entire deformation range. The energy density at a high strain rate was higher than that for the quasi-static or intermediate strain rate range at any strain level. From the previous theoretical analysis, the specimen deformation is nonuniform under the shock loading condition, where the front face of the specimen compresses rapidly and enters the densification regime and then this deformation moves to the next layer of the foam upon further compression. Such an effect combines the elastic, plateau, and densification effects in a small zone and does not lead to the appearance of individual zones in the energy density curve for the entire specimen. The final energy density with the high strain rate is 0.34 MJ/m^3^. Figure 11b shows the energy density for all the specimens. It can be seen that the energy density increases with the increase in the strain rate. The trends of energy density under high strain rate deformation in previous studies are similar to the results obtained in this test, as the energy density changed linearly with the increase in strain rate [54,55]. However, under a high strain rate, the energy density value varies in a wide range. This demonstrates that the energy density value is greatly affected by velocity under the shock mode.

### 4.4. Discussion

The novel test method based on a shock tube has advantages of long loading time and high loading rates, which are useful in investigating the impact properties of foams at high strain rates. Here, the test theory is presented, and the test platform is designed with a special foam fixture and a visualization of the end of the tube, which is able to test foam impact properties and monitor the foam failure progress. Based on the test method, the failure progress and final failure modes of PE foams are discussed and, moreover, energy absorption capabilities of PE foams are assessed under both quasi-static and high strain rate loading conditions in this paper. However, as the core of sandwich composites, the energy absorption of foam is still unclear. In addition, research on foam core sandwich composites will also be carried out by combining the foam material with a skin in further work.

## 5. Conclusions

This work investigated the energy absorption capability of closed-cell polyethylene foams under both quasi-static and high strain rate loading conditions. Through theoretical derivation, the calculation method of the energy absorption of foam material under shock mode deformation is solved. It provides a new calculation method for obtaining the energy absorption value of foam material. A new method for obtaining the stress–strain curve of foams under intermediate strain rate deformation is provided by modifying a drop weight test machine. Here, both a modified drop weight impact method and the shock tube test method were used for foam shock testing at high strain rates. The deformation and the strain of the foam were studied by a high-speed camera and, moreover, the energy absorption of the foam under different loading rates is discussed. The following conclusions can be drawn from this study:

1. In this study, the first and second transitional velocities of PE foam compression mode are calculated. The results of the theoretical model agree with the experimental results, which verifies the theory.
2. The difference in PE foam material behavior in homogeneous mode and shock mode was observed through experiments. The specimen deformation varies as the strain rate changes. In the shock mode, the stress in the specimen is not uniform. Shock tests resulted in strain rates of 6.1 × 10^3^ s^−1^, which were sufficiently high to cause selective densification in the proximal end of the specimen, as observed with a high-speed camera.
3. During the shock mode compression process, the energy absorption in the specimen varies linearly. When the speed is less than 1.3 m/s, this stable energy absorption state disappears. The energy density is greatly affected by velocity under the shock mode.

The above conclusion shows that the PE foam as a protective material for aircraft has good energy absorption characteristics. It can maximize its energy absorption characteristics and maintain stable energy absorption capacity under a high-speed impact.

## Data Availability

The data presented in this study are available on request from the corresponding author.
